# Mandibular trauma treatment: A comparison of two protocols

**DOI:** 10.4317/medoral.20263

**Published:** 2014-12-05

**Authors:** Paolo Boffano, Sofie C. Kommers, Fabio Roccia, Tymour Forouzanfar

**Affiliations:** 1 M.D, PhD student, Department of Oral and Maxillofacial Surgery/Pathology, VU University Medical Center and Academic Centre for Dentistry Amsterdam (ACTA), P.O. Box 7057, 1007 MB Amsterdam, The Netherlands; 2 M.D, Assistant Professor, Division of Maxillofacial Surgery, Head and Neck Department, San Giovanni Battista Hospital, University of Turin, Turin, Italy; 3 M.D. D.D.S. Ph.D, Head of Department and Professor, Department of Oral and Maxillofacial Surgery/Pathology, VU University Medical Center and Academic Centre for Dentistry Amsterdam (ACTA), P.O. Box 7057, 1007 MB Amsterdam, The Netherlands

## Abstract

Objectives: The aim of this study was to evaluate the treatment of mandibular fractures treated in two European centre in 10 years. Study Design: This study is based on 2 systematic computer-assisted databases that have continuously recorded patients hospitalized with maxillofacial fractures in two centers in Turin, Italy and in Amsterdam, the Netherlands for ten years. Only patients who were admitted for mandibular fractures were considered for this study. Results: Between 2001 and 2010, a total of 752 patients were admitted at Turin hospital with a total of 1167 mandibular fractures not associated with further maxillofacial fractures, whereas 245 patients were admitted at Amsterdam hospital with a total of 434 mandibular fractures. At Amsterdam center, a total of 457 plates (1.5 - 2.7 mm) were used for the 434 mandibular fracture lines, whereas at Turin center 1232 plates (1.5 – 2.5 mm) were used for the management of the 1167 mandibular fracture lines. At Turin center, 190 patients were treated primarily with IMF, whereas 35 patients were treated with such treatment option at Amsterdam center. Conclusions: Current protocols for the management of mandibular fractures are quite efficient. It is difficult to obtain a uniform protocol, because of the difference of course of each occurring fracture and because of surgeons’ experiences and preferences. Several techniques can still be used for each peculiar fracture of the mandible.

** Key words:**Mandibular fracture, facial trauma, maxillofacial, treatment, multicentre, database.

## Introduction

The maxillofacial region is one of the most frequently injured areas of the body, and in particular the mandible is the second most frequently fractured adult facial bone because of its prominent and unprotected position on the face (1-5).

Furthermore, mandibular fractures can cause a variety of impairments, including temporomandibular joint syndrome, poor mastication, dysocclusion, and chronic pain (1-5).

Treatment of these injuries is important to maintain speech, swallowing, and masticatory function. Treating mandibular fractures involves providing the optimal environment for bony healing to occur: adequate blood supply, immobilization, and proper alignment of fracture segments. As a result, most fractures require reduction and fixation to allow for primary or secondary bone healing (4).

The most common mandibular fracture varies according to centers and countries, with the condyle, angle or symphysis as the most frequently encountered fracture site (3-20).

Different treatment options for mandibular fractures have been described, including closed reduction and open reduction with fixation. Moreover, postoperative complications are related to the type of fracture, dislocation or displacement, and the chosen surgical treatment too.

Therefore, a thorough analysis of mandibular fracture treatment and outcomes is critical for the establishment of accurate trauma management protocols.

Continuous long-term collection of data regarding the treatment of mandibular fractures is important because it provides information necessary for the development and establishment of new algorithms and protocols of management of such injuries.

Therefore, the aim of this study was to evaluate the treatment and outcomes of mandibular fractures treated in two European centre in 10 years.

## Material and Methods

This study is based on 2 systematic computer-assisted databases that have continuously recorded patients hospitalized with maxillofacial fractures and surgically treated in the Division of Maxillofacial Surgery, San Giovanni Battista Hospital, Turin, Italy, and in the Department of Oral and Maxillofacial Surgery, Vrije Universiteit University Medical Center (VUMC), Amsterdam, the Netherlands, between January 1, 2001, and January 1, 2010.

Only patients who were admitted for mandibular fractures were considered for this study.

Patients affected by other associated fractures of the maxillofacial region and incomplete patient charts were excluded from this study in order to reduce bias and for the clarity of the data. Patients with dentoalveolar fractures were excluded too.

The following data for the injured patients were considered: sex, age, etiology, fracture site, treatment modality and complications.

The cause of injury was divided into six main categories: motor-vehicle accidents (MVA), which included accidents involving automobiles, motorcycles, and MVA - pedestrian accidents; assault, which included interpersonal violence and weapons attacks; falls; sport injuries; bicycle accidents; and (6) other causes, which included pathological fractures, occupational accidents, domestic accidents, suicide attempts, accidents with animals, tooth extraction, and unknown aetiology.

Patients were treated according to the departments’ protocol as demonstrated in  Table 1, Table 2, Table 3, Table 4.

**Table 1:**
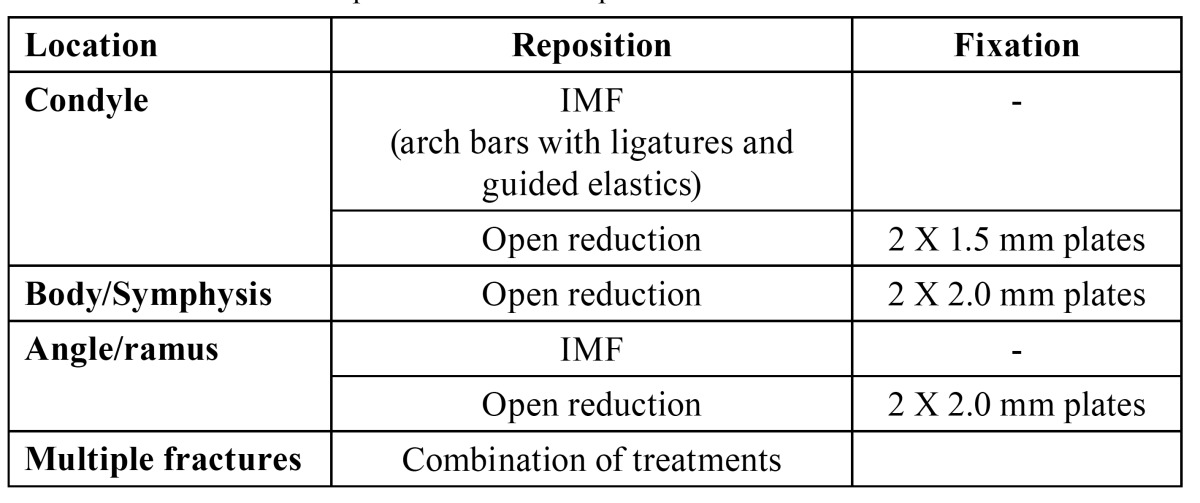
VUMC treatment protocol in dentate patients.

**Table 2:**
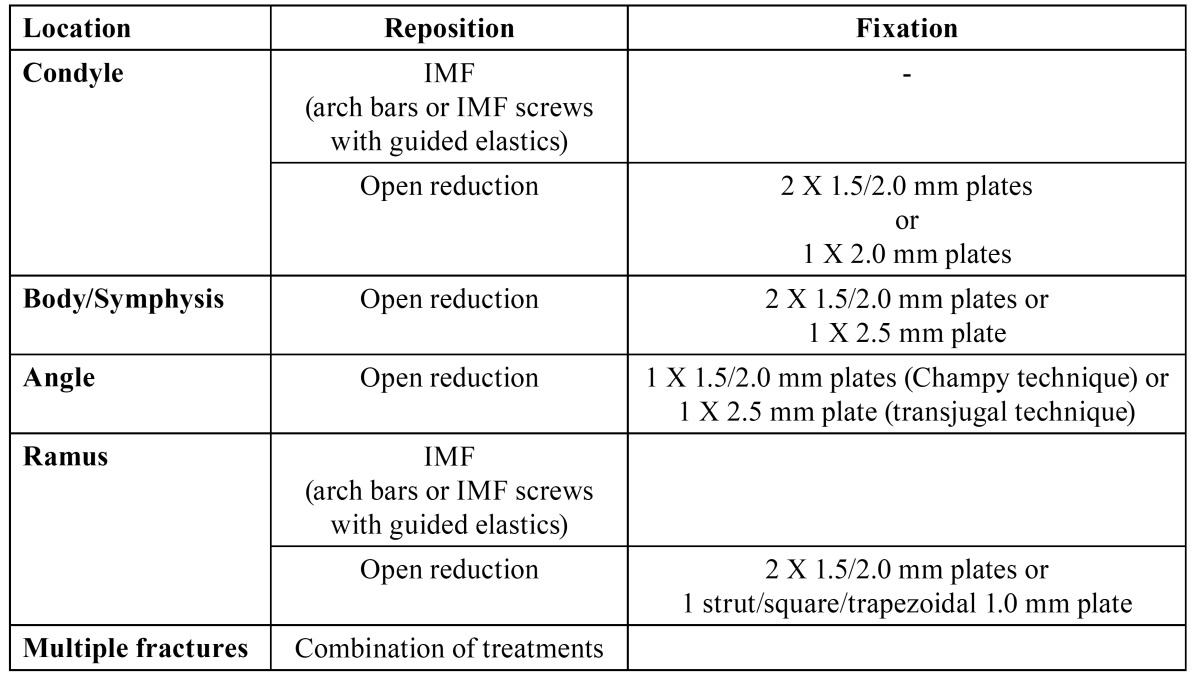
UNITO treatment protocol in dentate patients.

**Table 3:**
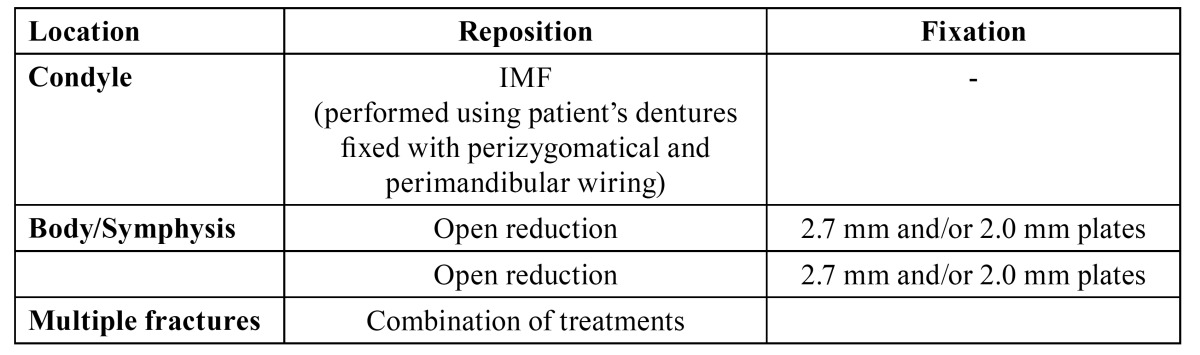
VUMC treatment protocol in edentulous patients.

**Table 4:**
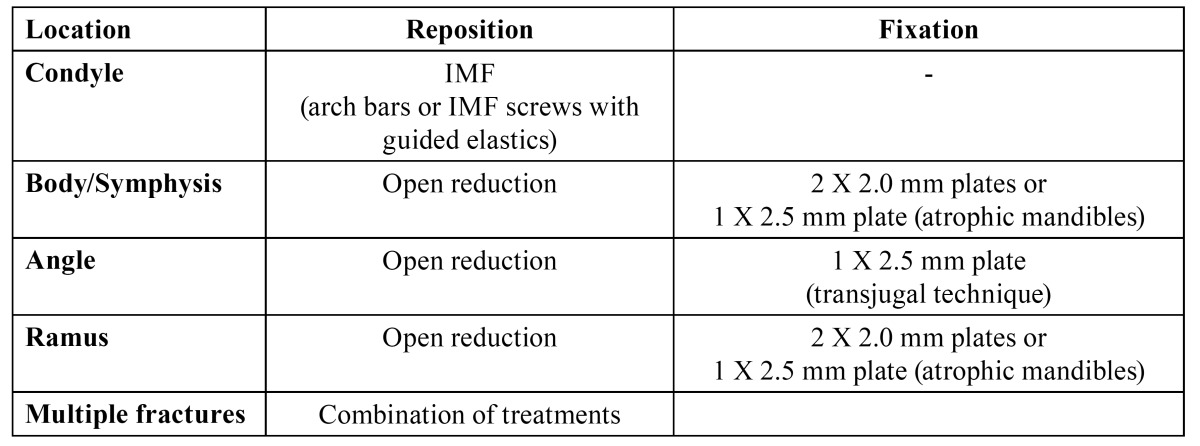
UNITO treatment protocol in edentulous patients.

As for condylar fractures, a closed treatment was performed in patients with condylar head fractures, non displaced subcondylar fractures, and condylar fractures in children. The remaining condylar fractures were treated by open reduction and internal fixation.

Antibiotic therapy was applied during the preoperative period. Prophylactic antibiotic therapy was applied in all cases, starting at the beginning of surgery during the intraoperative period. Antibiotics were routinely administered in the postoperative period. Post operatively all patients received standard analgesics (diclofenac 50 mg three times daily or paracetamol with codeine 1000/20 mg four times daily).

Post operatively conventional radiographs (panoramic radiograph) were performed to assess the reduction. If the reduction was performed sub optimally and there were clinical signs of a mandibular malunion, the patient was retreated. A strict follow-up to check return to standard mandibular function was prescribed to every patient for at least the first 6 postoperative weeks.

Osteosynthesis material was only removed in cases of persistent infection that did not respond to oral antibiotics (after 2-3 months post operatively), for age related reasons or for psychological reasons.

## Results

During the considered time frame, 1818 patients with maxillofacial fractures were admitted to the Division of Maxillofacial Surgery, San Giovanni Battista Hospital, Turin (UNITO), whereas 523 patients were admitted to the Department of Oral and Maxillofacial Surgery, Vrije Universiteit University Medical Center (VUMC), Amsterdam.

Between 2001 and 2010, a total of 752 patients (563 males, 189 females) were admitted at UNITO with a total of 1167 mandibular fractures not associated with further maxillofacial fractures, whereas 245 patients (169 males, 76 females) were admitted at VUMC with a total of 434 mandibular fractures.

The male/female ratio was 2.98:1 in UNITO-study population and 2.22:1 in VUMC case series.

In UNITO-study population a mean age of 34.8 years [range, 5 - 99; median, 30; standard deviation (SD), 18.5] was observed, in comparison with a mean age of 32 years (range, 2 - 87; median, 29; SD, 15.2) in VUMC patients.

The fractures were mainly the result of assaults (27% at VUMC, 29% at UNITO) in agreement with several articles in the recent literature (1-6), followed by falls (20% and 24%, respectively). Then, in VUMC study population bicycle accidents accounted for 20% of mandibular trauma, whereas in UNITO the third most frequent cause was represented by motor vehicle accidents (23%).

In both VUMC and UNITO, the most frequently involved fracture site was the mandibular condyle with 405 fracture lines (35%) in UNITO population and 185 fractures (43%) in VUMC series, followed by symphiseal/parasymphiseal fractures (respectively, 307 and 110 fracture lines). Then, in UNITO population, 301 (25%) angle fractures, 136 (12%) body fractures, 11 ramus fractures, and 7 coronoid fractures were encountered too. In VUMC series, 71 (17%) body fractures, 65 (15%) angle fractures, 2 ramus fractures, and 1 coronoid fractures were also observed.

At VUMC, out of 245 patients with mandibular fractures, 232 patients were dentate, whereas 13 were edentulous; a total of 457 plates (1.5 - 2.7 mm) were used for the 434 mandibular fracture lines.

Instead, at UNITO, out of 752 total patients, 689 patients were dentate whereas 63 were edentulous; a total of 1232 plates (1.5 - 2.5 mm) were used for the management of the 1167 mandibular fracture lines.

At UNITO center, 190 patients were treated primarily with IMF, whereas 35 patients were treated with such treatment option at VUMC.

In the VUMC series, 7 patients were treated with 2.7 mm plates for a comminuted body or angle fracture. These plates were applied transorally.

Instead, in the UNITO study population, a greater use of 2.5 mm plates was observed: 154 dentate or edentulous patients ( Table 2, Table 4) were treated by the fixation with a 2.5 mm plate for mandibular body, symphysis or angle fractures. In particular, 83 symphyseal or parasymphyseal fractures, 49 angle fractures, and 38 body fractures were fixed by a 2.5 mm plate in these patients. Such plates were placed by end oral, translesional, extra oral or transjugal approaches.

As for immediate postoperative complications, at VUMC, 37 patients complained of reduced sensitivity of the lip- and chin-region of the fractured side. After 6 months none of the patients complained of permanent hypoaesthesia, dysaesthesia or anaesthesia. Objective analyses were not performed.

On the whole, in the VUMC series, during follow-up 15 patients visited the outpatient clinic with a dysocclusion of whom 11 dysocclusions were corrected by traction through guided elastics; 2 patients were retreated surgically within 4 weeks post-operatively, one patient with a fractured mandibular body and another patient with a combined condyle fracture and a mandibular body. These patients underwent a revision of the reduction and fixation procedure. At VUMC, 6 patients presented with infected osteosynthesis material. In 3 of these patients the osteosynthesis material was removed. The remaining patients were successfully treated with oral antibiotics.

In the UNITO study population, 116 patients complained of inferior alveolar nerve dysesthesia, but 6 months follow-up information were not registered in the UNITO database. At UNITO, 6 patients were retreated surgically within 4 weeks post-operatively: 4 patients underwent reintervention because of postoperative dysocclusion, whereas 2 patients were surgically retreated because of a broken plate. Finally, in Turin study population, 12 plated were removed in the immediate postoperative period because of infection of the osteosynthesis site.

## Discussion

An understanding of the patterns and management of mandibular trauma is essential so that an effective prevention of injuries and efficient allocation of health care resources can be performed (1-9).

It is always crucial to record up-to-date information about mandibular fractures treatment and to compare it with other centers and with the literature. Furthermore, the multicentre collection of data, as in our study, will allow to obtain more reliable data with lower bias.

Fractures of the symphyseal and parasymphyseal region can generally be managed by lag screws or plates. In the VUMC and UNITO centers the use of plates for the fixation of such fractures is the preferred treatment. A plate is usually placed mono cortically at the tension band, paying attention not to damage the underlying tooth roots, whereas a second plate is placed at the inferior border. This two plates method is particularly useful in the parasymphyseal region where the near mental nerve has to be mobilized and retracted to allow for appropriate fixation (4). The two points of fixation are necessary to prevent rotational forces from causing the superior border from splaying and disrupting the continuity of the alveolar arch. Eventually, arch bars can also serve as the third tension band for the fractured region; however, it is not necessary if an appropriate fixation by plates has been performed. However, a one plate method can be adopted too: a thicker 2.5 mm or more plate can be sufficient in the symphyseal region to give stability to the fractured mandible. This option may have two weak points: the greater difficulty in modeling the plate and the higher risk of “plate feeling” by the patient in comparison with the 2 plates method.

Fractures in the mandibular body are generally managed via an intraoral approach by the placement of two mini plates, as in the symphyseal region.

Angle fractures pose a unique clinical challenge for reconstructive surgeons. In fact, no general consensus on the optimal treatment of mandibular angle fractures has been obtained. Current treatment protocols for angle fractures involve rigid fixation in conjunction with intraoperative inter maxillary fixation, that allows for absolute stability leading to primary bone union and permitting immediate limited postoperative physiological function (5).

The preferred methods in VUMC and UNITO centers are closed reduction and IMF and intraoral open reduction, internal fixation using a single 1.5 or 2.0 mm mini plate secured to the superior surface of the mandible (the Champy technique), and internal fixation by two 2.0 mm mini plates. In selected cases, where a higher immediate stability is needed, the open reduction and internal fixation using a 2.5 mm plate via a combined end oral / transjugal approach has been performed too.

Ramus fracture is an extremely rare injury. When the fracture is not displaced, a closed treatment with IMF can be used, whereas in patients with dislocated ramus fractures the protocols of VUMC and UNITO foresee an internal fixation by two 1.5/2.0 mm mini plates or by 1 strut/square/trapezoidal 1.0 mm plate in order to gain sufficient stability.

Finally, condylar fracture is the most challenging mandibular fracture and the wide and continue article production in the current literature (3-10) witness the importance of finding the highest consensus on the most appropriate management. At VUMC and UNITO centers, a closed treatment was performed in patients with condylar head fractures, non displaced subcondylar fractures, and condylar fractures in children. The remaining condylar fractures were treated by open reduction and internal fixation with two 1.5 or 2.0 mm mini plates if it was possible, or with a single mini plate if the level of the fracture or the dimension of the condyle did not allow the placement of 2 plates.

The low rate of complications observed in the two analyzed study populations demonstrates that current protocols for the management of mandibular fractures are quite efficient. It is difficult to obtain a uniform protocol, because of the difference of course of each occurring fracture and because of surgeons’ experiences and preferences. Several techniques can still be used for each peculiar fracture of the mandible, keeping in mind that primary stability and precocious postoperative function have now been acknowledged to be crucial for a rapid and complete recovery.

In conclusion, continuous long-term and multicentre collection of data about mandibular trauma treatment is important because it provides the information necessary for the development of multicentre protocols and consensus.
